# Syntaxin 5 Is Required for Copper Homeostasis in *Drosophila* and Mammals

**DOI:** 10.1371/journal.pone.0014303

**Published:** 2010-12-20

**Authors:** Melanie Norgate, Adam Southon, Mark Greenough, Michael Cater, Ashley Farlow, Philip Batterham, Ashley I. Bush, V. Nathan Subramaniam, Richard Burke, James Camakaris

**Affiliations:** 1 School of Biological Sciences, Monash University, Clayton, Victoria, Australia; 2 Genetics Department, University of Melbourne, Parkville, Victoria, Australia; 3 Mental Health Research Institute of Victoria, Parkville, Victoria, Australia; 4 Membrane Transport Laboratory, Queensland Institute of Medical Research, Brisbane, Queensland, Australia; Brunel University, United Kingdom

## Abstract

Copper is essential for aerobic life, but many aspects of its cellular uptake and distribution remain to be fully elucidated. A genome-wide screen for copper homeostasis genes in *Drosophila melanogaster* identified the SNARE gene *Syntaxin 5* (*Syx5*) as playing an important role in copper regulation; flies heterozygous for a null mutation in *Syx5* display increased tolerance to high dietary copper. The phenotype is shown here to be due to a decrease in copper accumulation, a mechanism also observed in both *Drosophila* and human cell lines. Studies in adult *Drosophila* tissue suggest that very low levels of *Syx5* result in neuronal defects and lethality, and increased levels also generate neuronal defects. In contrast, mild suppression generates a phenotype typical of copper-deficiency in viable, fertile flies and is exacerbated by co-suppression of the copper uptake gene *Ctr1A*. Reduced copper uptake appears to be due to reduced levels at the plasma membrane of the copper uptake transporter, Ctr1. Thus *Syx5* plays an essential role in copper homeostasis and is a candidate gene for copper-related disease in humans.

## Introduction

Copper (Cu) is essential to aerobic organisms as a cofactor in diverse metabolic processes including cellular respiration and proliferation; formation of connective tissue, melanin and neurotransmitters; antioxidant defence; cell signalling; and angiogenesis [1], [2]. Although several proteins involved in copper homeostasis have been well characterized, numerous aspects of the cellular distribution remain to be fully elucidated.

Ctr1 is the major copper uptake protein in mammalian cells, and is thought to form a trimer containing a pore at the plasma membrane through which copper can pass [3], [4]. Chaperones Atox1, CCS, Cox17, Sco1 and Sco2 are required to deliver copper to copper-dependent enzymes in various subcellular compartments. Atox1, CCS and Cox17 may receive their copper either directly via protein–protein interaction with Ctr1 or indirectly via an intermediate such as glutathione or metallothionein [5], and Sco1 has been shown to receive copper from Cox17 [6]. At the trans-Golgi network (TGN), copper is transferred from Atox1 directly to the transmembrane copper-translocating P-type ATPases ATP7A (MNK) and ATP7B (WND) for transport to enzymes of the secretory pathway [7]. However, under conditions of excess cellular copper, ATP7A and ATP7B traffic towards the plasma membrane where they facilitate copper efflux [8].

Due to the varied metabolic processes for which copper is required, there are a wide variety of copper-related diseases with diverse phenotypes. For example, Menkes disease is caused by impaired ATP7A-mediated transport of dietary copper from the polarised gut epithelial cells, resulting in systemic copper deficiency and Wilson disease is caused by impaired ATP7B-mediated transport of copper from the liver resulting in copper toxicosis [reviewed in 9]. However, not all copper-related diseases have been associated with a candidate gene [10]. Copper levels and copper metabolism proteins have been implicated in gene expression, tumour cell metastasis and resistance to anti-neoplastic drugs and copper chelators have shown promise in the treatment of cancer [reviewed in 2]. Copper dyshomeostasis in the brain is associated with Alzheimer's disease and copper ionophores have shown encouraging results in clinical trials [11]. The further characterisation of genes involved in copper homeostasis is therefore required to provide additional candidate genes and support our understanding of the mechanisms underlying a range of copper-related diseases [2].

The vinegar fly *Drosophila melanogaster* has recently proven a useful model for characterizing the role of copper homeostasis genes. *Drosophila* has orthologues of all major copper homeostasis proteins and several studies have demonstrated the high level of functional conservation with humans [12]–[15]. In *Drosophila*, two homologous proteins, Ctr1A and Ctr1B, fulfil the function of mammalian Ctr1. *Ctr1A* is constitutively expressed [13], [16] and is required for baseline copper uptake while *Ctr1B* is induced in the midgut by dietary copper limitation and is needed to boost absorption [13], [17]. DmATP7 is the sole *Drosophila* orthologue of mammalian copper transporting ATPases, ATP7A and ATP7B [15].

A genetic screen was performed in *Drosophila* to further illuminate our understanding of copper homeostasis mechanisms [18]. This resulted in the identification of the SNARE (soluble NSF attachment protein receptor) gene *Syntaxin 5* (*Syx5*) as playing an important role in copper regulation in the fly; flies heterozygous for a null mutation in *Syx5* display significantly increased tolerance to high levels of dietary copper.

SNAREs are involved in fusion of vesicles to target membranes and are therefore central to intracellular trafficking [19]. Syx5 is localised to the *trans*-Golgi network (TGN) for docking of vesicles including the COPI type [20] and is required for endosome to TGN transport of Shiga toxin and the endogenous cargo protein mannose 6-phosphate receptor [21]. There is also evidence for *Syx5*–mediated transport between endoplasmic reticulum (ER) and Golgi [22]. *Drosophila* Syx5 has recently been shown to play a role in translocation of proteins to the apical membrane and is also required for Golgi reassembly following cell division [23]. This signifies a degree of functional conservation of mammalian and *Drosophila* Syx5. Given the known roles of mammalian *Syx5* in both anterograde and retrograde intracellular trafficking, *Syx5* represents an excellent candidate for involvement in uptake or intracellular distribution of copper. The current study presents evidence that Syx5 is vital for efficient copper uptake in insect and mammalian cells as well as *in vivo* in *Drosophila*.

## Results

### *Syx5^+/−^* heterozygotes have high tolerance to dietary copper

**Figure 1:**
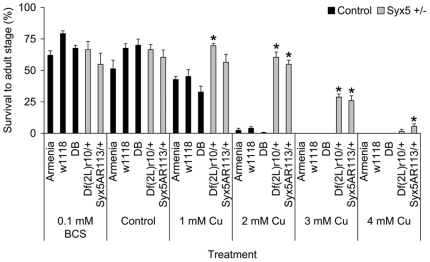
*Syx5* ^+/−^ *Drosophila* show increased dietary copper tolerance. ‘Armenia’ and ‘*w^1118^*’ are control strains with normal copper homeostasis mechanisms. ‘DB’ is a double balancer strain containing the *CyO* balancer chromosome present in the *Syx5^+/−^* mutants. ‘*Df(2L)r10*’ is an original mapping strain and ‘*Syx5^AR113^*’ is a specific *Syx5* null allele. Values are mean with s.e.m. Both *Syx5* heterozygous strains show increased copper tolerance compared to the three controls. *Significant difference from Armenia, determined by a Mann-Whitney test (P<0.05). There was no difference in survival between *w^1118^* and *Syx5^AR113/+^* flies on concentrations of BCS up to 1 mM (F = 0.778, P = 0.610).

Increased *Drosophila* copper tolerance had been previously mapped to a single locus on Chromosome 2 encoding *Syx5* [18]. To confirm the correct locus had been identified, *Syx5^AR113^/CyO Drosophila* were screened for copper tolerance (Fig. 1). The *Syx5^AR113^* allele encodes a functionally null, truncated peptide which is homozygous lethal [23]. The wild-type strain Armenia, the eye-colour mutant *w^1118^* and the mapping strain *Df(2L)r10, cn^1^/CyO*, with a deletion spanning *Syx5*, were included as controls. The offspring from crosses of *Syx5^AR113^/CyO*×Armenia, *Df(2L)r10*, *cn^1^/CyO*×Armenia, and a double-balancer stock (*IF/CyO*)×Armenia were also screened to confirm copper tolerance segregated with the *Syx5* mutations.

Both *Syx5^AR113^/CyO* and *Df(2L)r10, cn^1^/CyO* had significantly higher survival to the adult stage than Armenia, *w^1118^* or *IF/CyO* when reared on a copper-supplemented diet, but no difference in survival was observed on basal media or the copper chelator BCS (Figure 1). χ^2^ analysis of the crosses revealed that the increased copper tolerance segregates with both the *Df(2L)r10* deletion (χ^2^ = 26.385, P<0.001 on 1 mM Cu) and the *Syx5^A113^* allele (χ^2^ = 18.615, P<0.001), but not with any balancer or wild-type chromosomes (Table S1). This clearly demonstrates that increased copper tolerance in *Syx5^+/−^* heterozygotes is associated with a 50% reduction in Syx5 levels compared to wild-type *Drosophila*. The *Syx5^AR113^/CyO* strain was used to investigate how Syx5 mediates this copper tolerance. This strain shows no viability or fertility defects (Figure S1) indicating that the copper-related phenotypes demonstrated here are not due to a non-specific reduction in fitness.

### Copper tolerance is associated with reduced copper levels in *Syx5^+/−^ Drosophila*

**Figure 2:**
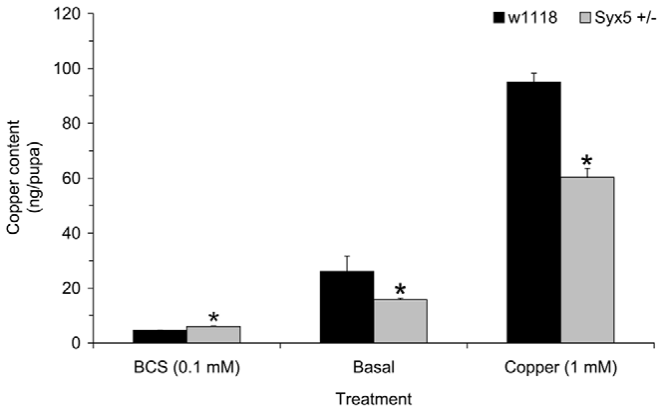
Copper accumulation in *Syx5* ^+/−^ *Drosophila*. Copper content was measured by ICP-AES in wild-type and *Syx5^+/−^ Drosophila* reared to the pupal stage on copper chelator (100 µM BCS), basal media or 1 mM copper. Values are mean metal content per pupa with s.e.m. from five replicates of 50 pupae. *Syx5^+/−^* larvae accumulate less copper on both basal and copper-supplemented media. *Significant difference from wild-type, determined by a Mann-Whitney test (P<0.05).

Pupal metal content was measured to ascertain copper accumulation throughout the larval feeding stage, as this is most relevant to the increased tolerance of dietary copper (Figure 2). *Syx5^+/−^* heterozygotes accumulate less copper than wild-type on both basal and copper-supplemented diets. This strongly suggests that the increased copper tolerance of *Syx5^+/−^* heterozygotes is due to reduced copper levels relative to wild-type flies although we cannot rule out alternative explanations for the copper tolerance phenotype with reduced copper content being an indirect consequence. Interestingly, they also accumulate more zinc than wild-type on a zinc-supplemented diet, but tolerance to excess zinc is unaffected (Figure S2). No other metals were significantly affected.

The induction of *metallothionein* genes has been used previously as a proxy marker for copper excess, and *Ctr1B* induction as a marker for copper deficiency [15]. These genes were examined in *Syx5^+/−^* larvae raised under basal and copper supplemented conditions (Figure S3). Higher expression of *Ctr1B* in *Syx5^+/−^* larvae compared to wild-type (3.2±0.7 times wild-type, independent samples T-Test, P<0.05) is consistent with copper deficiency under basal conditions [17], and is alleviated by copper-supplementation (1.1±0.1 times wild-type). Copper-supplemented media stimulated the normal *metallothionein* (copper sequestration) response, raising expression to similar levels in both *Syx5^+/−^* and wild-type larvae. *MtnC* was the only *metallothionein* to show lower expression compared to wild-type (0.1±0.0, P<0.05). These results are consistent with direct measurements of copper content (Figure 2) which show that *Syx5^+/−^* larvae are capable of accumulating copper on supplemented media, even though the levels do not reach those of wild-type. Higher concentrations (2–4 mM copper) result in a copper load sufficient to increase *Syx5^+/−^* mortality, despite the high tolerance compared to wild-type (Figure 1). Together these data indicate that flies with 50% wild-type levels of functional Syx5 accumulate excess copper, but do so less efficiently than wild-type flies.

### Tissue-specific reduction in Syx5 generates a typical copper deficiency phenotype

The GAL4-UAS system in *Drosophila* can be used to manipulate target gene expression in individual tissues by using tissue-specific GAL4 drivers to either ectopically express the gene of interest or inhibit it by RNA interference (RNAi) [24], [25]. RNAi lines specific to *Syx5* were used to suppress *Syx5* activity. Targeted suppression of *Syx5* in the developing eye (Gmr-GAL4), nervous system (Elav-GAL4) or midgut (Mex-GAL4) resulted in larval or pupal lethality, probably due to the essential role of this gene in intracellular trafficking and Golgi reassembly following cell division [23].

**Figure 3:**
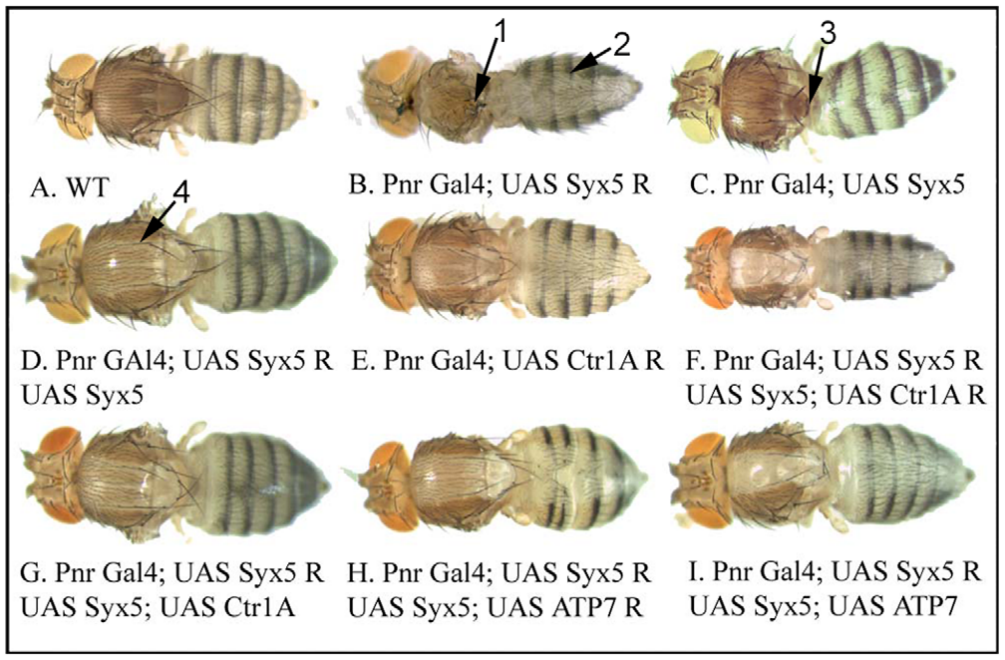
Suppression of *Syx5* in adult *Drosophila* cuticle results in hypopigmentation typical of copper deficiency. Gene expression/ suppression was driven in a dorsal stripe down the adult thorax/abdomen using the Pannier-GAL4 driver. A control fly is shown (A). *Syx5* suppression under Pnr-GAL4 is normally lethal. Rare survivors reared at 18°C (B) show loss of dorsal thorax (arrow 1) and strong abdominal hypopigmentation (arrow 2). *Syx5* over-expression results in reduced bristles and scutellum (C, arrow 3). *Syx5* suppression can be rescued to a mild hypomorph by concurrent *Syx5* over-expression (D), which shows hypopigmentation (arrow 4) similar to that seen for *Ctr1A* suppression (E). The mild hypomorph is exacerbated by *Ctr1A* co-suppression (F) but not rescued by *Ctr1A* over-expression (G). *DmATP7* suppression (H) and over-expression (I) phenotypes are unaffected by addition of the *Syx5* hypomorph combination.

Suppression of *Syx5* in the Pannier domain (Pnr-GAL4), a band down the centre of the developing thorax and abdomen, is also normally lethal. However rare survivors raised at 18°C show abdominal hypopigmentation phenotypes typical of copper deficiency [Figure 3B], [ 15,26] and complete loss of the central thorax. In contrast, over-expression of *Syx5* in the same domain resulted in reduced scutellum and bristles but no change in pigmentation (Figure 3C). This milder phenotype is not necessarily related to copper transport as bristles are a mechanosensory structure, so their loss can reflect neuronal defects [27].

Combining the *Syx5* suppression and over-expression transgenes in the same fly results in a moderate hypopigmentation of the thorax (Figure 3D) similar to that seen in the moderate copper deficiency caused by *Ctr1A* suppression [Figure 3E, 26]. This is most likely a hypomorphic *Syx5* phenotype where the combination of RNAi and over-expression results in an intermediate level of *Syx5* transcript causing a partial loss of function.

To investigate genetic interactions between Syx5 and the copper homeostasis machinery, *DmATP7* and *Ctr1A* levels were manipulated together with *Syx5* suppression. Over-expressing or suppressing either *Ctr1A* (copper uptake) or *DmATP7* (copper efflux) was unable to rescue the lethality caused by strong Syx5 suppression. Together with the *Syx5* hypomorph combination (Figure 3D), *Ctr1A* suppression is additive (Figure 3F); the hypopigmentation phenotype is more severe and there is bristle loss that is not observed for either *Ctr1A* suppression (Figure 3E) or in the *Syx5* (Figure 3D) hypomorph alone. In contrast *Ctr1A* over-expression, which would normally increase copper levels, does not rescue the *Syx5* hypomorph (Figure 3G). The *Syx5* hypomorph has no effect on the phenotype caused by either *DmATP7* suppression (Figure 3H) or over-expression (Figure 3I).

These studies demonstrate that strong suppression of *Syx5* causes lethality that cannot be rescued by the manipulation of major copper transporters which mediate uptake and efflux, indicating that intracellular trafficking pathways additional to those involved in copper homeostasis are disrupted. In contrast, mild suppression of *Syx5* leads to a typical copper deficiency phenotype in the adult thorax and abdomen that is exacerbated by suppression of *Ctr1A* and cannot be rescued by *Ctr1A* over-expression. The copper deficiency phenotype is consistent with a reduction in copper levels found in *Syx5^+/−^* heterozygous flies (Figure 2, Figure S3) and a disruption to copper transport (Figure 3E).

### *Syx5* suppression reduces copper uptake at the cellular level

**Figure 4:**
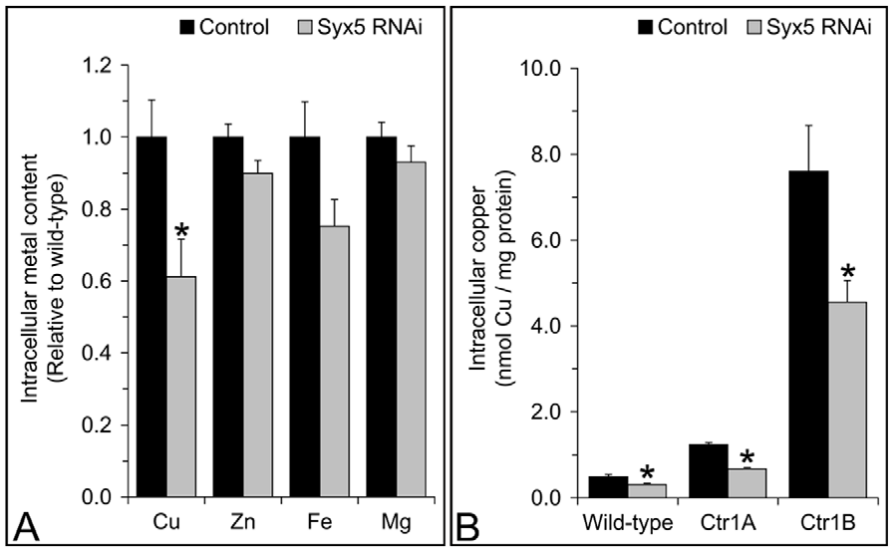
*Syx5* suppression in *Drosophila* S2 cells decreases copper accumulation. Metal accumulation was measured by ICP-AES in control (black) and *Syx5* (grey) RNAi suppression cells grown in basal media (A). *Syx5* gene expression was suppressed to 19–36% of wild-type levels. Values are mean with s.e.m of eight replicates over two experiments, normalized against control cells. Mean copper accumulation during a 24 h exposure to 2 µM Cu was measured using ^64^Cu in S2 cell lines stably over-expressing *Ctr1A*, *Ctr1B* or an empty vector control and normalized to total cellular protein (B). Error bars are s.e.m. from nine replicates over three experiments. *Syx5* gene expression levels relative to wild-type were 18–41% (control cells), 25-41% (Ctr1A) and 23-31% (Ctr1B). Copper accumulation is reduced by *Syx5* suppression (A) even when *Ctr1A* or *Ctr1B* is over-expressed (B): suppression of *Syx5* reduces copper levels to 50–70% of wild-type in all cell lines. *Significant difference between control and *Syx5* suppression cells, determined by an independent samples T-Test (P<0.05).

Copper accumulation was studied in cultured cells to further examine the role of Syx5 in cellular copper homeostasis. Suppression of *Syx5* in the *Drosophila* S2 embryonic cell line resulted in reduced copper accumulation (Figure 4A), consistent with the reduction seen in pupae (Figure 2). No other metals were significantly affected. In particular, in contrast to the whole animal inductively coupled plasma atomic emission spectrometry (ICP) results, zinc content was not altered by *Syx5* suppression in cells. A reduction in copper levels appears to be the key cellular *Syx5* suppression phenotype and was therefore examined further.

To investigate how *Syx5* might affect copper homeostasis, the gene was suppressed in cells with stable over-expression of *Ctr1A* or *Ctr1B* (Figure 4B). While total copper accumulation was higher in cells over-expressing either copper uptake gene, the relative efficiency of accumulation was decreased to a similar extent in control and over-expression cell lines when *Syx5* was suppressed. This is consistent with data from *Syx5^+/−^* heterozygote flies, which show they are able to accumulate excess copper, but do so less efficiently than wild-type flies (Figures 1–2, Figure S3).

**Figure 5:**
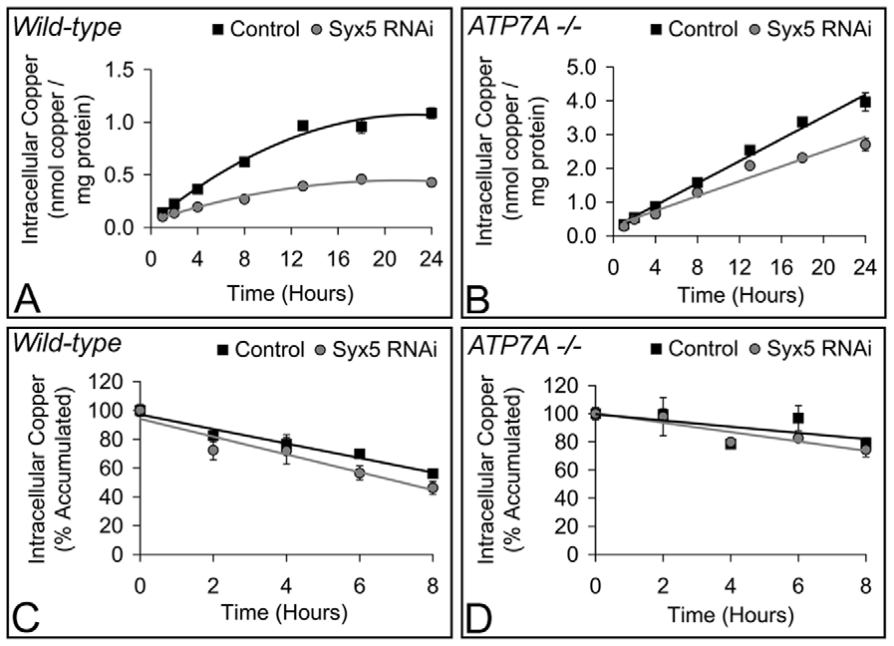
*Syx5* suppression in human cells decreases copper accumulation but does not affect rate of turnover. Wild-type (GM2069; A) and ATP7A deficient (Me32a; B) human cell lines were exposed to control (squares) or *Syx5* (circles) siRNA for 48 h. *Syx5* gene expression levels relative to wild-type were 22–46% for GM2069, and 23n45% for Me32a cells. Copper accumulation was then measured with ^64^Cu following 1–24 h exposure to 2 µM copper. Values are mean with s.e.m. of six replicates over two experiments. Non-linear regression analysis demonstrated copper accumulation in GM2069 cells (A) was significantly reduced following suppression of *Syx5* (F = 108.0, P<0.0001). Linear regression analysis demonstrated copper accumulation was also significantly reduced in Me32a cells (B) following suppression of *Syx5* (F = 44.1, P<0.0001). Rate of copper turnover of the radioisotope ^64^Cu was measured in wild-type (GM2069; C) and ATP7A deficient (Me32a; D) human cell lines. Cells were treated with control (squares) or *Syx5* (circles) siRNA and exposed to 2 µM copper for 24 h, then returned to basal media for 2–8 h. Data are expressed as a percentage of copper accumulation at Time 0 and expressed as mean with s.e.m. of nine replicates over three experiments. Linear regression analysis shows that the rate of copper turnover was not significantly altered by *Syx5* suppression.

The effect of human *Syx5* suppression was also investigated in two human fibroblast cell lines (Figure 5 and Figure S4). GM2069 cells have wild-type copper transport mechanisms. Me32a cells were derived from a Menkes disease patient and have a deletion in the *ATP7A* gene that introduces a premature stop codon [28]. These cells hyper-accumulate copper as the truncated ATP7A protein is unable to facilitate efflux of excess copper and the ATP7B efflux protein is not expressed.

Copper accumulation was determined at time intervals from 1 to 24 h (Figure 5A–B). Suppression of *Syx5* resulted in a significant reduction in copper accumulation in both cell lines, with the difference most evident in GM2069 cells between 13 and 24 h when copper levels plateau. These results are consistent with those in S2 cells exposed to copper following suppression of *Syx5* (Figure 4). Suppression of *Syx5* does not significantly affect the rate of copper uptake in GM2069 cells up to 1 h (Figure S4A) or the short term copper uptake kinetics (Figure S5B–C). The reduced copper accumulation in Me32a cells (Figure 5B) indicates that functional ATP7A is not required for the *Syx5* suppression phenotype. This suggests that the copper deficiency phenotype is associated with copper uptake rather than efflux, consistent with gene interaction studies in *Drosophila* (Figures 3 and 4B).

To exclude the possibility that *Syx5* suppression stimulates an ATP7A-independent efflux mechanism, cells were allowed to accumulate copper and copper retention was examined when cells were returned to basal media (Figure 5C–D). Although less total copper accumulated following suppression of the gene (Figure 5A–B), there was no effect on the rate of copper turnover (Figure 5C–D) indicating increased copper efflux is unlikely to be responsible for the reduction in copper accumulation. Taken together these results demonstrate that suppression of *Syx5* reduces copper uptake efficiency when cells are exposed to copper in the micromolar range. Reduced copper accumulation is evident after 1 h and the greatest difference occurs when copper levels have reached a steady state.

**Figure 6:**
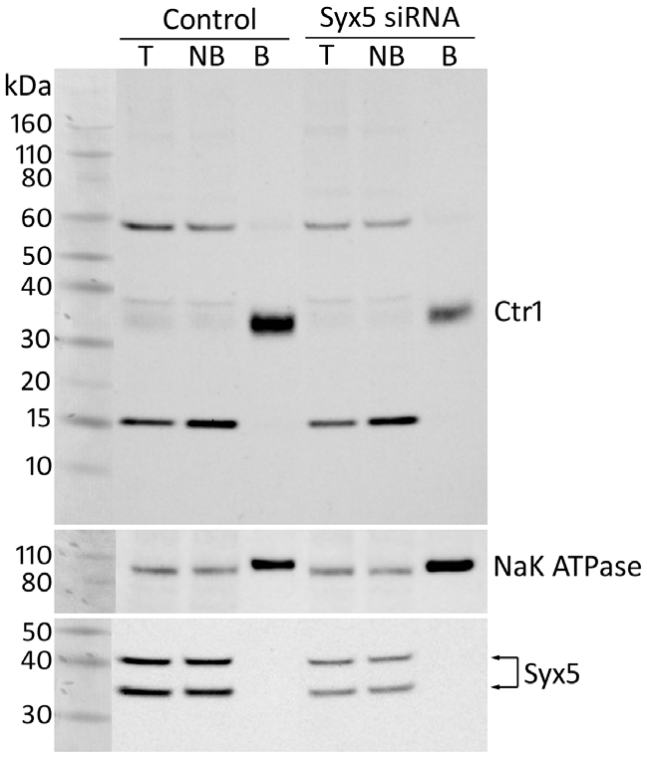
*Syx5* suppression reduces the amount of hCtr1 at the plasma membrane of Hek293 cells. Hek293 cells stably expressing Ctr1-myc were treated with scrambled negative control or Syx5 siRNA. Cell surface proteins were isolated following biotinylation using biotin-streptavidin precipitation prior to western immuno-blotting. Ctr1was detected with an anti-c-myc antibody. Syx5 knockdown was confirmed with an anti-Syx5 antibody and anti-NaK ATPase was used as control. Total lysate (T), non-biotinylated (NB), and biotinylated (B) fractions are shown. Protein bands were quantified with densitometry. Two species of Syx5 were detected and Syx5 knockdown reduced the amount of this protein in total cell lysate to approximately 33% of control siRNA treated cells. An hCtr1 monomer of approximately 35 kDa was detected in the biotinylated fraction. Densitometric analysis of hCtr1 protein intensity relative to NaK ATPase, revealed that Syx5 knockdown reduced the about of hCtr1 at the cell surface to approximately 20% of control siRNA treated cells.

The localisation of human Ctr1 (hCtr1) was examined in human embryonic kidney (HEK293, ATCC cell line CRL-1573) cells following *Syx5* suppression using biotinylation to detect myc-tagged hCtr1 at the cell surface (Figure 6). A biotinylated protein of approximately 35 kDa was detected at the cell surface, comparable to the reported size of monomeric myc-tagged hCtr1 in HEK293 cells [29]. *Syx5* suppression reduced the amount of hCtr1 detected at the plasma membrane whilst a control membrane transporter, NaK-ATPase was not affected. Densitometry analysis revealed that, relative to NaK-ATPase, hCtr1 levels at the cell surface were reduced to 20% of that in control cells, consistent with the finding that copper uptake is reduced.

Western blot densitometric analysis showed that Syx5 levels were reduced to 24–33% of wild-type in the human cell lines (Figures 6 and S5). This is milder suppression than in cases where Golgi fragmentation has been reported [21], [30] and minimal disruption to the Golgi and early endosomes was observed here (Figure S6). This is consistent with results from *Drosophila* in suggesting that copper homeostasis phenotypes are observed in otherwise viable, fertile flies when Syx5 levels are only mildly reduced. Greater reductions cause additional cellular disturbances and a range of phenotypes not necessarily related to copper homeostasis.

## Discussion

Roles for mammalian Syx5 in both anterograde and retrograde vesicular transport have been well characterized and severe Syx5 reduction causes fragmentation and dispersal of the Golgi [21], [30]. Similarly, the *Drosophila Syx5* orthologue is required for Golgi reassembly following cell division and for translocation of proteins to the apical membrane [23]. Complete loss of Syx5 activity leads to early larval lethality in the fly [23], as does the strong targeted RNAi suppression achieved by most GAL4 drivers tested for this study.

In contrast, results presented here have shown that a 50% reduction in Syx5 levels in *Drosophila* leads to significantly increased tolerance to high dietary copper with no adverse impact on viability or fertility. *Drosophila* copper tolerance can be altered by manipulating levels of known copper uptake proteins or metallothioneins directly or via suppression of the metallothionein transcription factor, MTF-1 [12], [13], [31]. However, the present study implicates for the first time a gene encoding components of vesicular trafficking in copper tolerance. Direct measurement of copper levels and analysis of copper-induced gene expression showed that this increased tolerance is associated with a reduction in copper accumulation. Consistent with this, moderate suppression of *Syx5* specifically in the adult thorax generated a typical copper-deficiency phenotype.

Reduced Syx5 levels also resulted in elevated zinc accumulation in the fly but, unlike the copper phenotype, this did not affect zinc sensitivity. A possible explanation is that, with reduced copper levels, more metallothionein is available to sequester excess zinc and the flies are able to absorb more without detrimental effects. It is notable that this change in zinc accumulation was seen in *Drosophila* pupae only, not in cell lines, and could therefore be a systemic response to reduced copper accumulation.

The study of copper uptake and retention in cultured cells confirmed that the *in vivo* copper deficiency phenotype is due to a reduction in the efficiency of copper accumulation. Importantly, consistent results were seen in both *Drosophila* and human cell lines, suggesting that Syx5 plays an evolutionarily conserved role in cellular copper uptake. In human cell lines, we observed no detectible effect on copper uptake kinetics over 10 minutes, but rather a gradual reduction in copper accumulation over a period of hours. Copper turnover was unaffected by suppression of *Syx5* and copper levels differed most at steady state levels.

Consistent with the finding that Syx5 suppression affects the copper uptake pathway, hCtr1 levels at the plasma membrane were reduced to 20% of control when Syx5 was suppressed in human cells. Previous studies have found that flies lacking *Drosophila* Syx5 have impaired transport of proteins to the apical membrane of epithelial cells [23]. Due to barely detectable Ctr1 levels in the whole lysate, it cannot be determined whether overall Ctr1 levels are reduced in these cells. Given the known role of Syx5 in anterograde vesicle transport and apical protein targeting, the most likely explanation is that Syx5 is required for localization of Ctr1 to the plasma membrane. However alternative explanations such as reduced synthesis or stability of Ctr1 cannot be ruled out. Thus it appears that loss of Syx5 alters Ctr1 function, thus inhibiting cellular copper uptake. This leads to a systemic copper deficiency *in vivo*.

Although mild dispersion of the Golgi was seen in Syx5 suppression cells and it has been shown previously that the loss of Syx5 can cause severe cellular and fertility defects [23], the impairment to copper uptake observed here occurred in the presence of sufficient Syx5 that flies show normal viability and fertility compared to wild type flies. Since the copper-tolerant *Syx5^+/−^* heterozygotes examined here are otherwise healthy, this raises the possibility that mild loss of Syx5 function may be important in copper-related disease in humans. This may be particularly relevant to conditions such as cancer and Alzheimer's disease, where subtle changes in cellular copper regulation may influence progression of the disease [1], [2], [11]. Indeed, it has been found that defects in components of the trafficking machinery can lead to a specific disease phenotype [32], although this has not previously been documented for copper homeostasis. In the case of Alzheimer's disease adapter proteins can affect Abeta40 and Abeta42 production by altering residence time of amyloid precursor protein in particular compartments including the plasma membrane [33].

We have presented evidence for a key role of Syx5 in cellular copper uptake, indicating that it plays a significant role in copper homeostasis. The finding that mild loss of Syx5 function significantly influences intracellular copper levels in the absence of other obvious phenotypes at the whole organism level provides a novel candidate for etiology of diseases resulting from copper dyshomeostasis [e.g. 2], [10].

## Materials and Methods

### *Drosophila* stocks and maintenance

All *Drosophila* strains were maintained on standard medium at 25°C. Armenia, *Arm^60^* (European *Drosophila* Stock Centre, Umeå Sweden). *w^1118^* (BL3605, Bloomington Stock Centre). ‘Df(2L)r10’, *Df(2L)r10*, *cn^1^/CyO* (BL1491). *Syx5^AR113^/CyO*, also referred to as *Syx5^+/−^* (BL3645) [23]. ‘Double balancer’, *w; IF/CyO; MKRS/TM6b, Tb* (gift from G. Hime, University of Melbourne). Gmr-GAL4, *P{Gmr-GAL4.w^−^}2* (BL9146). Mex-GAL4 [34]. Pnr-GAL4, *P{GawB}pnr^MD237^* (BL3039). ‘UAS-Syx5’, RNAi transformants 3857 and 3859 specific for *Syx5* yielded the same results, and data shown are for 3857 (Vienna *Drosophila* RNAi Center).

### *Drosophila* mortality screen

Standard medium was supplemented with 100–1000 µM of the copper chelator bathocuproinedisulfonic acid (BCS; Sigma) or 1–4 mM Cu (CuSO_4_.5H_2_O; Merck) as specified in the text. Survival to the adult stage was measured for five replicates of 50 first instar larvae per condition. Male *Syx5^AR113^*/CyO, *Df(2L)r10*/CyO or *IF/CyO* mutants were crossed to female Armenia and visible Chromosome 2 markers were used to monitor segregation of *Syx5^AR113^*, *Df(2L)r10* and *CyO*. Offspring from the replicates were pooled for χ^2^ analysis.

### Transgenics

The *Drosophila Syx5* open reading frame including the first intron was PCR amplified from *w^1118^* genomic DNA, omitting the termination codon using primers: Forward, GGGGTACCATGCAAACCCGAAGACGCCT and Reverse, GCTCTAGACGACATAAAAACAACGAAG. This fragment was sub-cloned in-frame with a C-terminal myc epitope tag into the pUAST_attB vector. Embryos from the Basler laboratory φC31 strains φX-51A and φX-96E were injected by standard techniques. Microinjections utilized an Eppendorf Femtojet apparatus with Femtotips II (Eppendorf) pre-pulled glass needles. Integrants at both these attP sites were obtained. Results presented here utilized the φX-51A integrant. Adult flies were imaged with a Leica MZ6 Stereomicroscope.

### Generation of myc-tagged Crt1 overexpressing HEK293 cells

The myc-tagged Ctr1 construct was generated through PCR amplification of cDNA using the forward (5′-TCATGGATCCGAAAAAATGGAACAAAAACTCATCTCAGAAGAGGATCTGGATCATTCCCACCATATGGG -3′) and reverse (5′-GGGCTCTAGAGAATTCAATGGCAATGCTCTGTGATATC -3′) oligonucleotides and by incorporation into the mammalian expression vector, pcDNA3. The forward oligonucleotide introduced sequence encoding the myc epitope in-frame immediately after the start codon and provided a flanking 5′ *Bam*H1 endonuclease restriction site. The reverse oligonucleotide provided an *Eco*RI endonuclease restriction site 3′ to the stop codon. Template cDNA was isolated from human hepatoma HepG2 cells (ATCC, cell line HB-8065) using the SuperScriptTM III CellsDirect cDNA synthesis system (Invitrogen) following the manufacturer's protocol. The PCR reaction contained 1×PCR buffer, 0.2 mM of each dNTP, 2 mM MgCl_2_, 0.2 µM of each primer, 2.5 units Platinum Taq DNA polymerase and 3 µl of cDNA (Invitrogen). Reactions were run on an Eppendorf Epgradient S Mastercycler on the following program: one cycle of 94°C for 2min, 38 cycles of 94°C for 45s, 57°C for 60s and 72°C for 60 s, followed by one cycle of 72°C for 2 min. The resultant PCR product was digested with *Bam*H1 and *Eco*R1 and cloned into pcDNA3 at the same sites. Integrity was confirmed by sequencing. Stable transfection of HEK293 cells (ATCC, cell line CRL-1573) with the myc-tagged Ctr1 construct was performed using FuGENE® HD (Roche) following the manufacturer's instructions. The cells were recovered in Dulbecco's Modified Eagle's medium (DMEM) containing 10% (v/v) FCS and transfectants were selected with 500 µg/ml G418 for 14 days.

### Cell culture

*Drosophila* embryonic S2 cells were propagated in Serum Free Media (SFM, Invitrogen) as previously reported [14]. S2 cells maintaining stable over-expression of *Ctr1A* or *Ctr1B* were generated by co-transfecting pCoHygro with either pAcCtr1A, pAcCtr1B or pAc empty vector control using Lipofectamine2000 and propagated in Schneider's Complete Media (Invitrogen) with 10% foetal calf serum (Trace Scientific) supplemented with 300 µg/ml hygromycin-B according to the manufacturer's instructions (Invitrogen). Media was replaced with SFM for all experiments and supplemented with CuCl_2_ (Sigma) at the concentration specified in the text. Wild-type (GM2069) and ATP7A null (Me32a) human fibroblast cells have been described previously [28]. Cells were maintained in Eagle's basal culture medium (Thermo Scientific) supplemented with 10% foetal calf serum (Trace Scientific) at 37°C and passaged weekly. Human embryonic kidney (HEK293) cells were stably transfected with myc-tagged human Ctr1 (pcDNA3.1Ctr1-Myc). These cells were maintained in Dulbecco's modified Eagle's medium (Thermo Scientific) with 10% foetal calf serum (Trace Scientific) and 500 µg/ml Geneticin (Invitrogen) at 37°C and passaged weekly.. All experiments were conducted in growth media with 10% foetal calf serum.

### RNA interference and gene expression

dsRNAi in S2 cells was conducted as previously reported [14]. dsRNA was targeted to *Syx5* (cDNA bases 214–757). Control dsRNA was derived from EYFP cDNA or *Adult Cuticle Protein 1* (13–558), which is not expressed in S2 cells. siRNA suppression in mammalian cells utilized Stealth RNAi duplexes (Invitrogen). 40 nM *Syx5* or low GC negative control Stealth RNAi duplexes were transfected into mammalian cells with Lipofectamine2000 according to the manufacturer's instructions (Invitrogen). Cells were seeded to be 30–50% confluent on the day of transfection and growth media was replaced with Opti-MEM (Invitrogen). Opti-MEM was replaced with growth media 4–6 h after transfection 48 h before experiments. Gene suppression was confirmed using qPCR and western blot. qPCR was performed as previously described [31]. Housekeeping genes *GAPDH*, *Actin42A* and *βActin* were used for normalisation in *Drosophila* larvae, *Drosophila* S2 cells and mammalian cells respectively. Primer sequences are shown in Table S2.

### Metal accumulation and retention

Copper accumulation was measured as previously reported [14]. Cells were incubated with ∼0.4 MBq ^64^Cu (Australian Radioisotopes) and non-radioactive copper at the concentrations described in the figure legends. Copper retention was measured by incubating cells with copper for 24 h, washing, and incubating for an additional 2–8 h in basal media. Radioactivity was measured with a γ-counter (1282 CompuGamma, LKB Wallac). Copper levels were normalized to total cellular protein, which was determined using BioRad protein reagent according to the manufacturer's instructions (BioRad). Total metal accumulation was measured using a Vista-AX Inductively Coupled Plasma Atomic Emission Spectrometer (ICP-AES, Varian) in samples digested in 70% HNO_3_ and metal levels were measured as described previously [31]. *Drosophila* metal levels were measured in five replicates of 50 pupae and expressed as ng/pupa. S2 cell metal levels were normalized to total cellular protein.

### Cell Surface Biotinylation and Western Blotting

Cell surface proteins were labelled with 0.5 mg/ml sulpho-NHS-SS-biotin (Thermo Scientific) and precipitated with streptavidin-agarose beads (Thermo Scientific) as previously described [35]. Protein samples were resolved on NuPAGE 4–12% Bis-Tris gels (Invitrogen) and transferred to nitrocellulose membranes for western immuno-blotting. Primary antibodies used were mouse anti-c-myc (1∶5000, Sigma), rabbit anti-Syx5 [1∶1500, 36] and mouse anti-NaK-ATPase (1∶5000, Abcam). Horseradish peroxidase coupled secondary antibodies were rabbit anti-mouse and goat anti-rabbit (1∶7000, Dako). Chemiluminescence was detected using ECL (GE Healthcare) and images were captured with a Fujifilm LAS-3000 (Fujifilm LifeScience). Densitometric analysis was conducted with Multi Gauge v2.3 (Fujifilm).

### Statistics

Statistical analysis was conducted using SPSS v16 (SPSS). A one-sample Kolomogorov-Smirinov test was used to assess whether data was normally distributed. Statistical analyses are described in Figure legends. P<0.05 was deemed statistically significant.

## Supporting Information

Table S1 χ^2^ values comparing siblings from crosses to wild-type Armenia.
(0.03 MB DOC)

Table S2 Quantitative PCR primer sequences.
(0.03 MB DOC)

Figure S1 *Syx5^+/−^ Drosophila* show no viability or fertility defects. Ten individual pairs each of *Syx5^+/−^* heterozygous virgin females and *w^1118^* males (Syx5 f×w1118 m), *w^1118^* virgin females and *Syx5^+/−^* heterozygous males (w1118 f×Syx5 m), or *w^1118^* virgin females and *w^1118^* males (w1118×w1118) were maintained in vials containing standard laboratory medium which was replaced every 24 h. Fertility was measured by allowing pairs 24 h to mate, then counting eggs produced every 24 h for five days (A). Male reproductive output was measured as the egg production of females inseminated by *Syx5^+/−^* males. Twenty replicates of 50 eggs were transferred into vials containing standard laboratory medium and viability was scored as the number of adults to emerge after 20 days (B). The average number of eggs laid per female over a five day period and the average number of eggs to reach the adult state were compared among strains using a one-way ANOVA with LSD post-hoc testing. Since both *Syx5^−^/w^1118^* and *CyO/w^1118^* offspring were produced in *Syx5^+/−^* crosses, an independent samples T-Test was used to confirm there was no difference in survival between these sibling genotypes then they were pooled for comparison to the *w^1118^* strain. The *Syx5* mutation did not adversely affect fertility: there was no significant difference in the number of eggs produced from either cross compared to those produced by the *w^1118^* control strain (*Syx5^+/−^* female×*w^1118^* male, 306±15; *w^1118^* female×*Syx5^+/−^* male, 241±24; *w^1118^* female×*w^1118^* male, 295±32; P = 0.479). There was no adverse impact on viability of eggs from *Syx5^+/−^* parents, in fact there was slightly higher survival of the *w^1118^* female×*Syx5^+/−^* (44±1 s.e.m.) male compared to the *w^1118^* strain (38±1; P = 0.002). Emergence from the reciprocal cross was intermediate (40±1) and not significantly different from either.
(0.14 MB TIF)

Figure S2 Metal accumulation and zinc tolerance in *Syx5^+/−^* heterozygote *Drosophila*. Metal content was measured by ICP-AES on flies reared to the pupal stage on basal media (A). Data are mean ± s.e.m. metal content per pupa from five replicates of 50 pupae and are expressed relative to wild-type (*w^1118^*) levels. In addition to a decrease in copper accumulation (shown in detail in Figure 2 of the main text), *Syx5^+/−^* flies accumulated 1.5-fold more zinc than wild-type (A). Zinc tolerance was determined as described for copper tolerance in the main text by supplementing media with 0–8 mM zinc (ZnSO_4_.7H_2_0, Ajax) (B). No significant differences in mortality were detected.
(0.37 MB TIF)

Figure S3 Copper-responsive gene expression in *Syx5^+/−^ Drosophila*. Wild-type and *Syx5^+/−^ Drosophila* were reared to third instar on basal media (A) or 1 mM copper (B) and qPCR was used to investigate *Ctr1B* and *MtnA-D* expression levels from three replicates of 50 larvae. *Ctr1A* has no transcriptional response to copper levels and is included as a control. Gene expression is mean relative to wild-type. Error bars are s.e.m. Under basal conditions *Ctr1B* is upregulated in *Syx5^+/−^* larvae, indicative of copper deficiency. Copper exposure alleviates the deficiency and leads to similar *MtnA*, *MtnB* and *MtnD* upregulation in *Syx5^+/−^* and wild-type larvae. An independent samples T-Test was used to determine statistical significance for differences between *Syx5^+/−^* and wild-type exceeding a two-fold magnitude, as indicated by dotted lines (*P<0.05).
(0.24 MB TIF)

Figure S4 *Syx5* suppression in human cells does not significantly affect copper uptake kinetics. (A) Copper uptake measured over one hour in GM2069 cells treated with control (squares) or *Syx5* (circles) siRNA for 48 h. ^64^Cu was used to measure copper accumulation in cells exposed to 2 µM copper for 5–60 minutes. Values are mean with s.e.m. of nine replicates from three experiments. There was a tendency for the rate of copper accumulation to be lower following *Syx5* suppression, however linear regression analysis demonstrated that this was not significantly different. (B) GM2069 cells treated with control (squares) or *Syx5* (circles) siRNA for 48 h. ^64^Cu was used to measure copper accumulation in cells exposed to 2–100 µM copper for 10 min. Values are mean with s.e.m. of six replicates from two independent experiments. Non-linear regression analysis demonstrated that copper uptake kinetics were not statistically significant different following knockdown of *Syx5*.
(0.31 MB TIF)

Figure S5 RNAi suppression in human cells reduces protein levels for *Syx5*. GM2069 and Me32a cells treated with control or *Syx5* siRNA for 48 h. Western blot analysis of whole cell lysate from these cells using anti-Syx5 antibody detected two electrophoretic species as previously reported [1]. Rabbit anti-Actin 20–33 (1∶300, Sigma) was used as a loading control. The amount of both Syx5 species was reduced by *Syx5* suppression in each of these cell lines: Densitometry analysis demonstrates that, relative to control cells, Syx5 protein levels were reduced to 27.7% in GM2069 and 31.1% in Me32a cells. 1. Subramaniam VN, Loh E, Hong WJ (1997) N-ethylmaleimide-sensitive factor (NSF) and alpha-soluble NSF attachment proteins (SNAP) mediate dissociation of GS28-syntaxin 5 Golgi SNAP receptors (SNARE) complex. *J Biol Chem* 272: 25441–25444.
(0.24 MB TIF)

Figure S6 *Syx5* suppression does not cause substantial Golgi fragmentation or affect early endosome localization in human cells. *Golgi distribution:* Immunocytochemistry in GM2069 cells utilized anti-Syx5 (1∶50) and mouse anti-Golgin 97 was used as a TGN marker (1∶200, Prof. Paul Gleeson). Secondary antibodies were Alexa 488 anti-rabbit and Alexa 594 anti-mouse (1∶400, Invitrogen). DAPI (300 nM, Invitrogen) was used to detect the nucleus. Images were recorded at 100× magnification using an Olympus FluoView 1000 confocal microscope with Olympus FluoView ver1.6a software (Olympus). Images at each wavelength were captured sequentially and multi-color maximum brightness stacked images were prepared using Image J (NIH, Bethesda, MD, USA). GM2069 cells were treated with control siRNA (A–C) or *Syx5* siRNA (D–F). Syx5 is shown in Green (A, D), Golgin 97 is shown in Red (B, E) and DAPI is shown in Blue. Merged images are also shown (C, F). *Syx5* suppression reduced Syx5 levels but did not dramatically alter the distribution of Golgin 97 (D–F). Golgi distribution was measured using Image J and was found to be 42.5±7.2 µm^2^ in control and 72.2±9.7 µm^2^ following *Syx5* suppression. Thus the *Syx5* RNAi suppression achieved in this study produced a mild phenotype in comparison to the effects of extreme *Syx5* inhibition found in previous studies [1], [2]. *Early endosome localization:* Immunocytochemistry in GM2069 cells utilized anti-Syx5 (1∶50) and mouse anti-EEA1 was used as an early endosome marker (1∶100, BD Biosciences). Secondary antibodies, DAPI staining and image analysis were conducted as described above for Golgi distribution. GM2069 cells were treated with control siRNA (G–I) or *Syx5* siRNA (J–L). Syx5 is shown in Green (G, J), EEA1 is shown in Red (H, K) and DAPI is shown in Blue. Merged images are also shown (I, L). The localization of early endosomes was not affected by *Syx5* suppression (J–L). 1. Amessou M, Fradagrada A, Falguieres T, Lord JM, Smith DC, Roberts LM, Lamaze C, Johannes, L (2007) Syntaxin 16 and syntaxin 5 are required for efficient retrograde transport of several exogenous and endogenous cargo proteins. *J Cell Sci* 120: 1457–1468. 2. Diao A, Frost L, Morohashi Y, Lowe M (2008) Coordination of Golgin Tethering and SNARE Assembly: GM130 binds Syntaxin 5 in a p115-regulated manner. *J Biol Chem* 283: 6957–6967.
(0.84 MB TIF)
