# Supplementary material for: Syntaxin 5 Is Required for Copper Homeostasis in Drosophila and Mammals
Source: PLoS One. 2010 Dec 20;5(12):e14303. doi: 10.1371/journal.pone.0014303 (PMC3004795; doi:10.1371/journal.pone.0014303)
Supplement: Table S1 — χ2 values comparing siblings from crosses to wild-type Armenia. (0.03 MB DOC) [file pone.0014303.s001.doc]

Table S1. χ^2^ values comparing siblings from crosses to wild-type Armenia.

|  | DB (*IF,Cy*) x Armenia | *Df(2L)r10* x Armenia | *Syx5^AR113/+^* x Armenia |
| --- | --- | --- | --- |
| 0.1 mM BCS | 0.716 | 2.160 | 0.021 |
| Control | 3.571 | 6.919^†^ | 3.272 |
| 1 mM Cu | 1.220 | 26.385^**^ | 18.615^**^ |
| 2 mM Cu | 1.000 | 15.696^**^ | 12.000^*^ |

*,***Syx5^+/-^* greater than *Syx5^+/+^* emergence, *P < 0.01 and **P < 0.001; ^†^*Syx5^+/-^* less than *Syx5^+/+^* emergence, P < 0.01.
