# Supplementary material for: Syntaxin 5 Is Required for Copper Homeostasis in Drosophila and Mammals
Source: PLoS One. 2010 Dec 20;5(12):e14303. doi: 10.1371/journal.pone.0014303 (PMC3004795; doi:10.1371/journal.pone.0014303)
Supplement: Table S2 — Quantitative PCR primer sequences. (0.03 MB DOC) [file pone.0014303.s002.doc]

Table S2. Quantitative PCR primer sequences

| **Gene** | **Sense primer** | **Antisense primer** |
| --- | --- | --- |
| *Drosophila* larvae: | | |
| *Ctr1A* | GCTGGAATATCGACCTGTGA | ATCGAAGGTGGTTGCTTGT |
| *Ctr1B* | AGCAGCGTAGGAAGAACGA | CAGGGACTGGACGATGTG |
| *MtnA* | CCTGCAACTGCGGATCT | CGCAGGCGGATTTCTT |
| *MtnB* | ATGGTTTGCAAGGGTTGTG | TTGCAGGCGCAGTTGT |
| *MtnC* | CAAAGGCTGCGGAACAA | GCACTTGCAGTCCTGATTACAG |
| *MtnD* | AGTGCTCCGCCACCAA | TGTCCTTGGGTCCGTTCT |
| *GAPDH* | CCACTGCCGAGGAGGTCAACTA | GCTCAGGGTGATTGCGTATGCA |
| *Drosophila* S2 cells: | | |
| *Syx5* | GATGGTCGCCAGGTTTATTG | GTCGGGCAATTTGTTGATTT |
| *Actin42A* | gcttcgctgtctactttcca | cagcccgactactgcttaga |
| Mammalian cells: | | |
| *Syx5* | tggcacacatggttaaggaa | gcaaggaagaccacaaagatg |
| *βActin* | GATTCCTATGTGGGCGACGAG | CCATCTCTTGCTGGAAGTCC |
