# Supplementary figures and images for: Syntaxin 5 Is Required for Copper Homeostasis in Drosophila and Mammals
Source: PLoS One. 2010 Dec 20;5(12):e14303. doi: 10.1371/journal.pone.0014303 (PMC3004795; doi:10.1371/journal.pone.0014303)

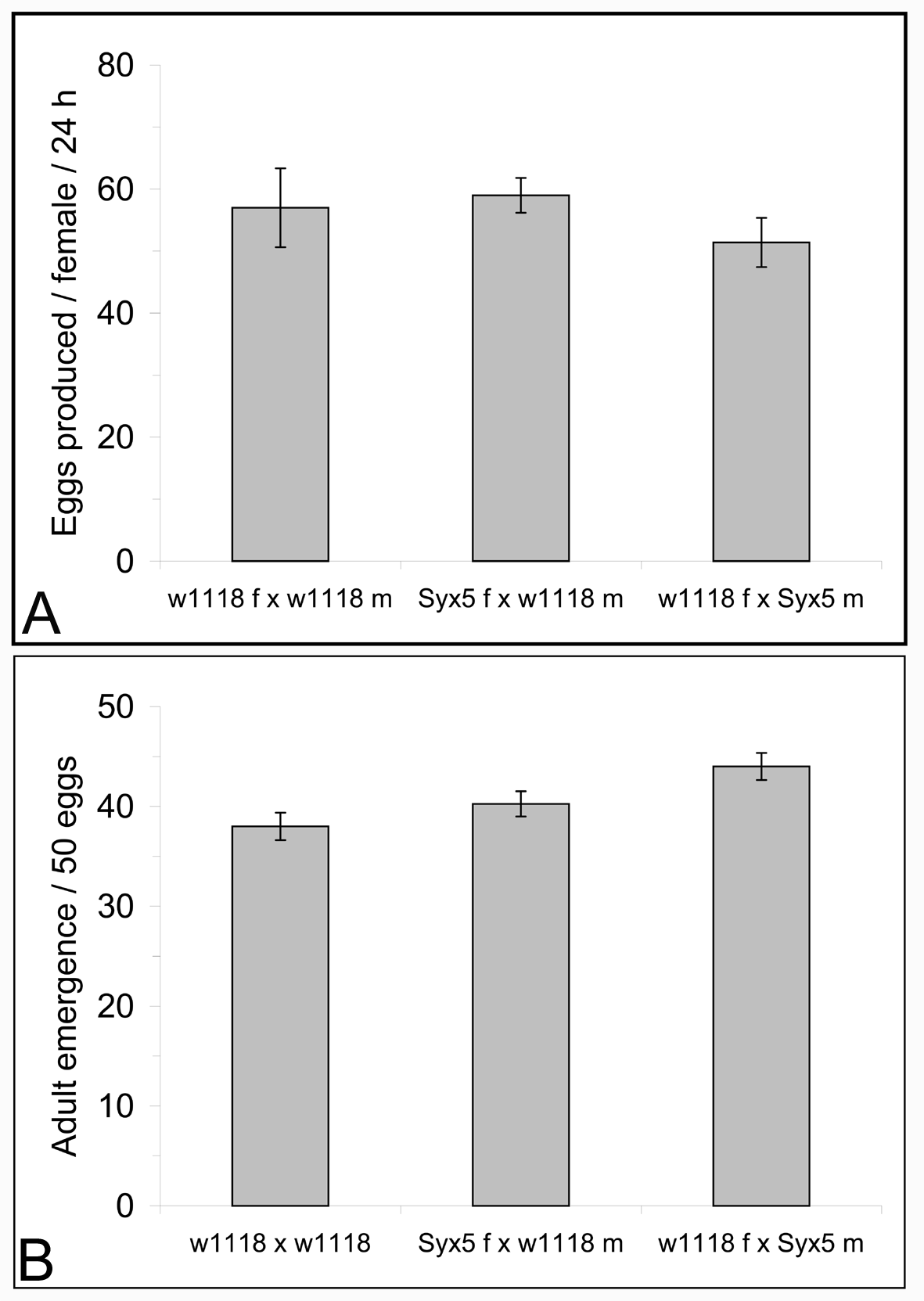

Supplement: Figure S1 — Syx5+/− Drosophila show no viability or fertility defects. Ten individual pairs each of Syx5+/− heterozygous virgin females and w1118 males (Syx5 f×w1118 m), w1118 virgin females and Syx5+/− heterozygous males (w1118 f×Syx5 m), or w1118 virgin females and w1118 males (w1118×w1118) were maintained in vials containing standard laboratory medium which was replaced every 24 h. Fertility was measured by allowing pairs 24 h to mate, then counting eggs produced every 24 h for five days (A). Male reproductive output was measured as the egg production of females inseminated by Syx5+/− males. Twenty replicates of 50 eggs were transferred into vials containing standard laboratory medium and viability was scored as the number of adults to emerge after 20 days (B). The average number of eggs laid per female over a five day period and the average number of eggs to reach the adult state were compared among strains using a one-way ANOVA with LSD post-hoc testing. Since both Syx5−/w1118 and CyO/w1118 offspring were produced in Syx5+/− crosses, an independent samples T-Test was used to confirm there was no difference in survival between these sibling genotypes then they were pooled for comparison to the w1118 strain. The Syx5 mutation did not adversely affect fertility: there was no significant difference in the number of eggs produced from either cross compared to those produced by the w1118 control strain (Syx5+/− female×w1118 male, 306±15; w1118 female×Syx5+/− male, 241±24; w1118 female×w1118 male, 295±32; P = 0.479). There was no adverse impact on viability of eggs from Syx5+/− parents, in fact there was slightly higher survival of the w1118 female×Syx5+/− (44±1 s.e.m.) male compared to the w1118 strain (38±1; P = 0.002). Emergence from the reciprocal cross was intermediate (40±1) and not significantly different from either. (0.14 MB TIF) [file pone.0014303.s003.tif]

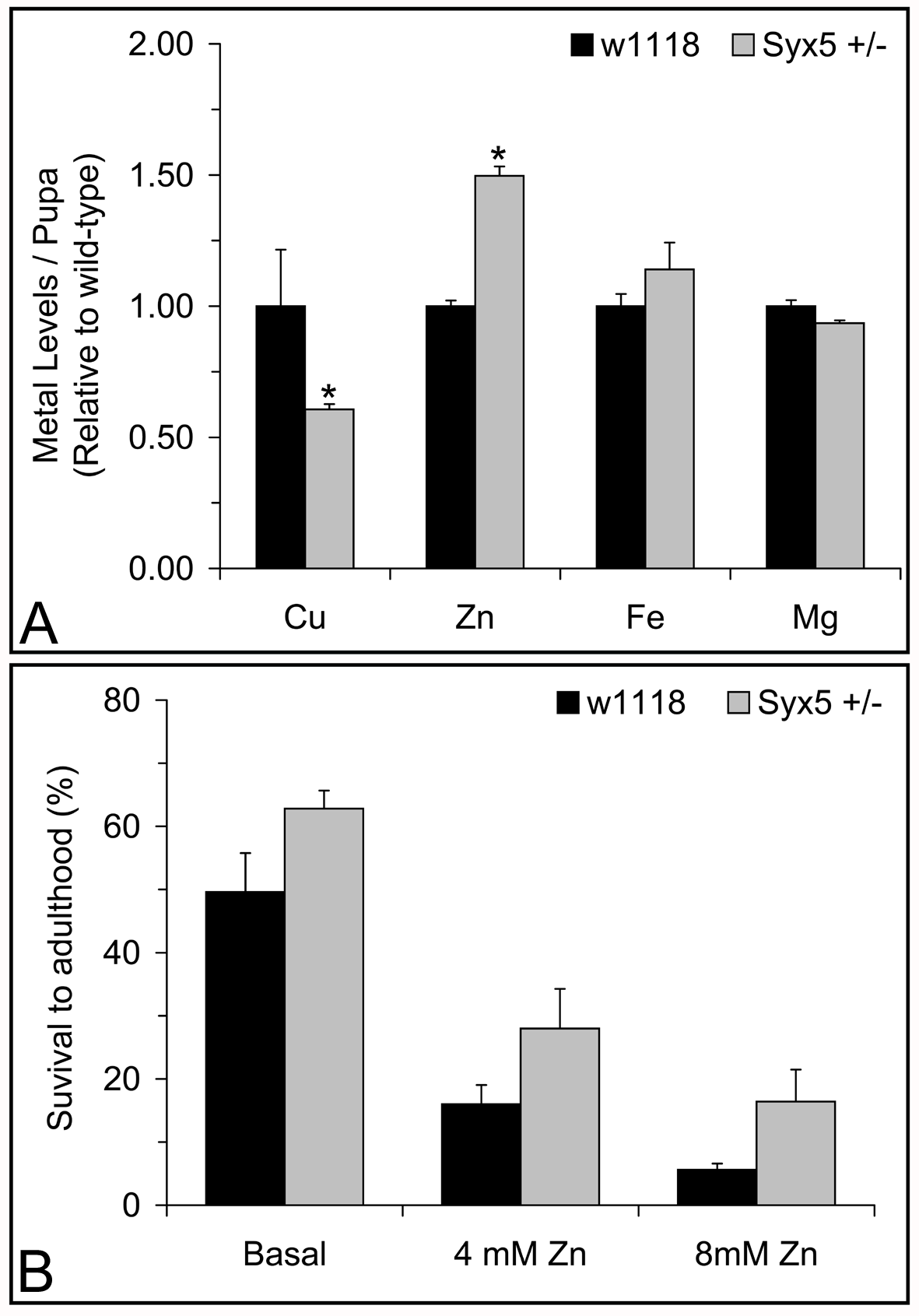

Supplement: Figure S2 — Metal accumulation and zinc tolerance in Syx5+/− heterozygote Drosophila. Metal content was measured by ICP-AES on flies reared to the pupal stage on basal media (A). Data are mean ± s.e.m. metal content per pupa from five replicates of 50 pupae and are expressed relative to wild-type (w1118) levels. In addition to a decrease in copper accumulation (shown in detail in Figure 2 of the main text), Syx5+/− flies accumulated 1.5-fold more zinc than wild-type (A). Zinc tolerance was determined as described for copper tolerance in the main text by supplementing media with 0–8 mM zinc (ZnSO4.7H20, Ajax) (B). No significant differences in mortality were detected. (0.37 MB TIF) [file pone.0014303.s004.tif]

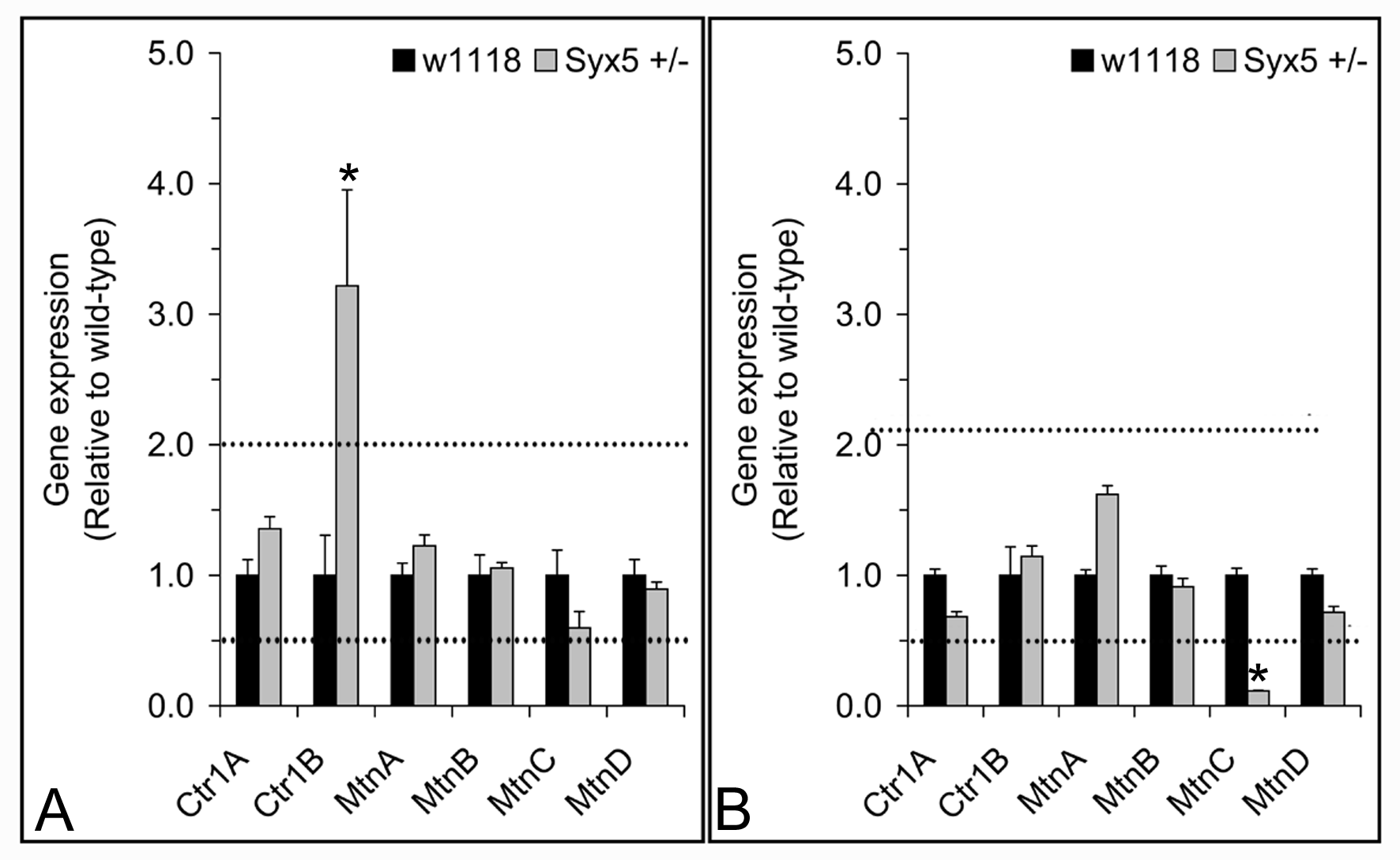

Supplement: Figure S3 — Copper-responsive gene expression in Syx5+/− Drosophila. Wild-type and Syx5+/− Drosophila were reared to third instar on basal media (A) or 1 mM copper (B) and qPCR was used to investigate Ctr1B and MtnA-D expression levels from three replicates of 50 larvae. Ctr1A has no transcriptional response to copper levels and is included as a control. Gene expression is mean relative to wild-type. Error bars are s.e.m. Under basal conditions Ctr1B is upregulated in Syx5+/− larvae, indicative of copper deficiency. Copper exposure alleviates the deficiency and leads to similar MtnA, MtnB and MtnD upregulation in Syx5+/− and wild-type larvae. An independent samples T-Test was used to determine statistical significance for differences between Syx5+/− and wild-type exceeding a two-fold magnitude, as indicated by dotted lines (*P<0.05). (0.24 MB TIF) [file pone.0014303.s005.tif]

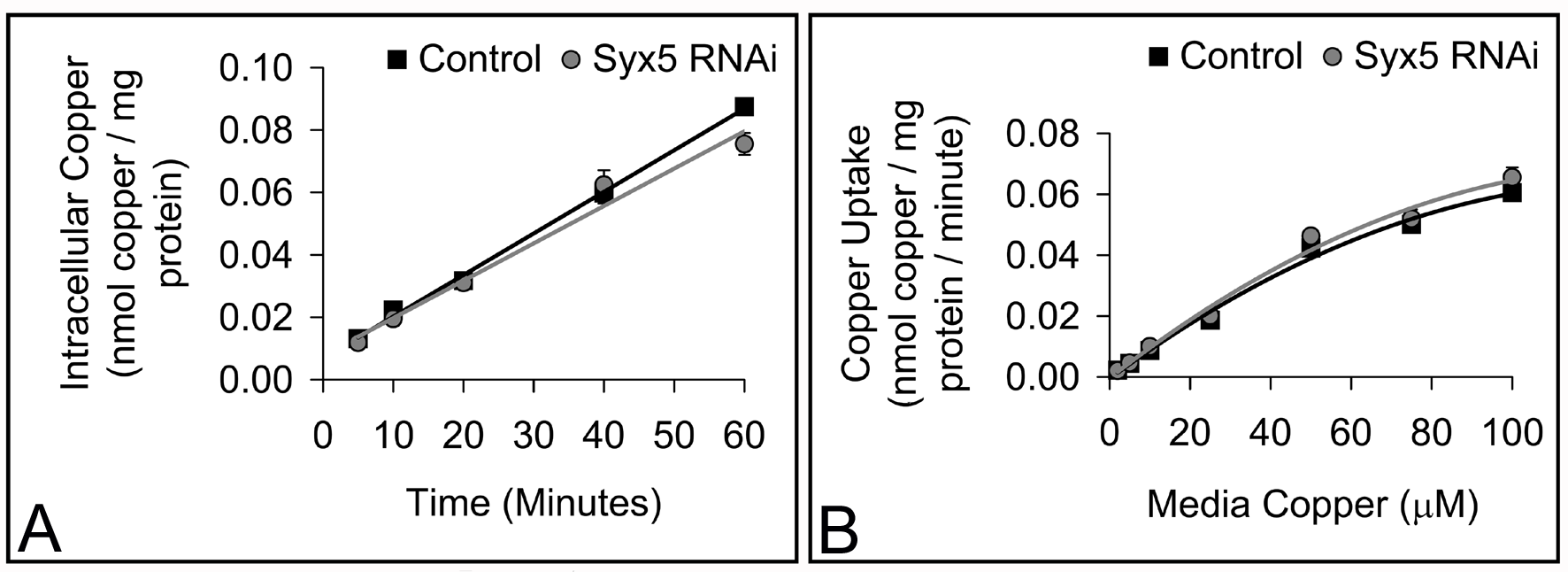

Supplement: Figure S4 — Syx5 suppression in human cells does not significantly affect copper uptake kinetics. (A) Copper uptake measured over one hour in GM2069 cells treated with control (squares) or Syx5 (circles) siRNA for 48 h. 64Cu was used to measure copper accumulation in cells exposed to 2 µM copper for 5–60 minutes. Values are mean with s.e.m. of nine replicates from three experiments. There was a tendency for the rate of copper accumulation to be lower following Syx5 suppression, however linear regression analysis demonstrated that this was not significantly different. (B) GM2069 cells treated with control (squares) or Syx5 (circles) siRNA for 48 h. 64Cu was used to measure copper accumulation in cells exposed to 2–100 µM copper for 10 min. Values are mean with s.e.m. of six replicates from two independent experiments. Non-linear regression analysis demonstrated that copper uptake kinetics were not statistically significant different following knockdown of Syx5. (0.31 MB TIF) [file pone.0014303.s006.tif]

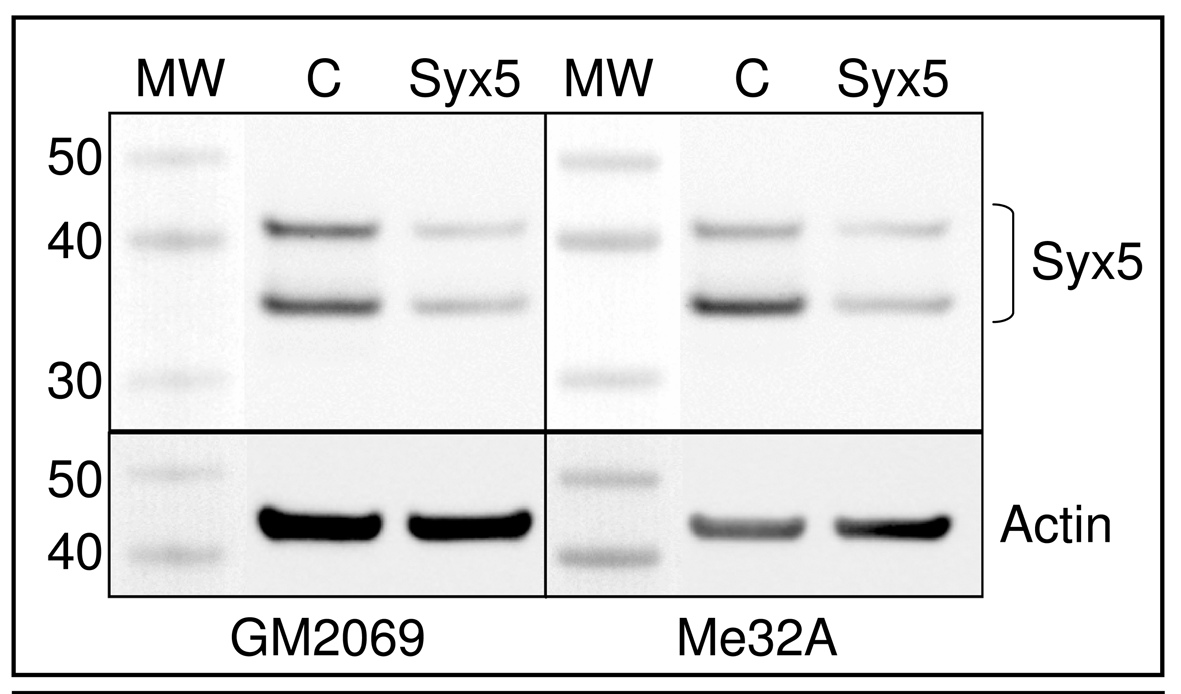

Supplement: Figure S5 — RNAi suppression in human cells reduces protein levels for Syx5. GM2069 and Me32a cells treated with control or Syx5 siRNA for 48 h. Western blot analysis of whole cell lysate from these cells using anti-Syx5 antibody detected two electrophoretic species as previously reported [1]. Rabbit anti-Actin 20–33 (1∶300, Sigma) was used as a loading control. The amount of both Syx5 species was reduced by Syx5 suppression in each of these cell lines: Densitometry analysis demonstrates that, relative to control cells, Syx5 protein levels were reduced to 27.7% in GM2069 and 31.1% in Me32a cells. 1. Subramaniam VN, Loh E, Hong WJ (1997) N-ethylmaleimide-sensitive factor (NSF) and alpha-soluble NSF attachment proteins (SNAP) mediate dissociation of GS28-syntaxin 5 Golgi SNAP receptors (SNARE) complex. J Biol Chem 272: 25441–25444. (0.24 MB TIF) [file pone.0014303.s007.tif]

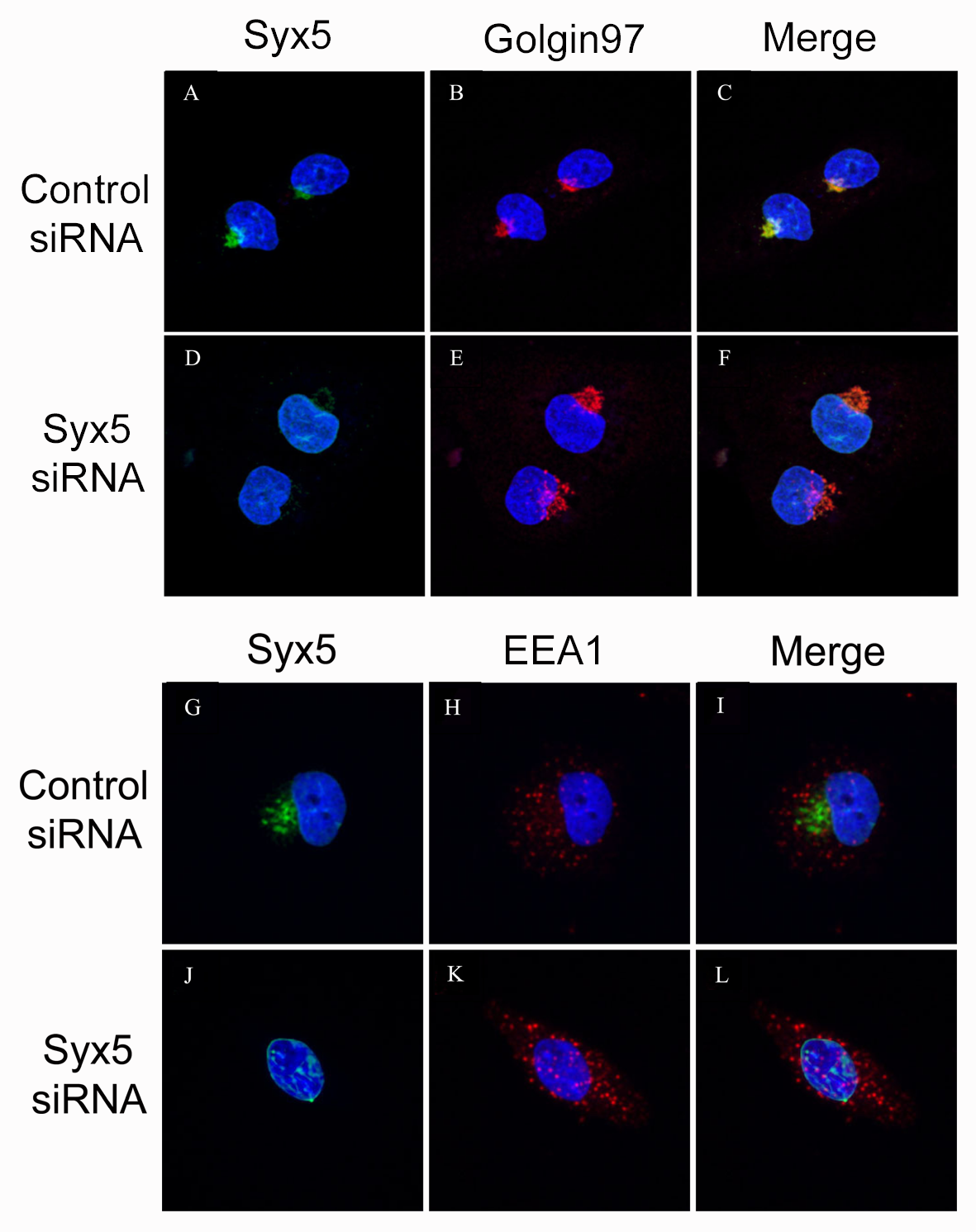

Supplement: Figure S6 — Syx5 suppression does not cause substantial Golgi fragmentation or affect early endosome localization in human cells. Golgi distribution: Immunocytochemistry in GM2069 cells utilized anti-Syx5 (1∶50) and mouse anti-Golgin 97 was used as a TGN marker (1∶200, Prof. Paul Gleeson). Secondary antibodies were Alexa 488 anti-rabbit and Alexa 594 anti-mouse (1∶400, Invitrogen). DAPI (300 nM, Invitrogen) was used to detect the nucleus. Images were recorded at 100× magnification using an Olympus FluoView 1000 confocal microscope with Olympus FluoView ver1.6a software (Olympus). Images at each wavelength were captured sequentially and multi-color maximum brightness stacked images were prepared using Image J (NIH, Bethesda, MD, USA). GM2069 cells were treated with control siRNA (A–C) or Syx5 siRNA (D–F). Syx5 is shown in Green (A, D), Golgin 97 is shown in Red (B, E) and DAPI is shown in Blue. Merged images are also shown (C, F). Syx5 suppression reduced Syx5 levels but did not dramatically alter the distribution of Golgin 97 (D–F). Golgi distribution was measured using Image J and was found to be 42.5±7.2 µm2 in control and 72.2±9.7 µm2 following Syx5 suppression. Thus the Syx5 RNAi suppression achieved in this study produced a mild phenotype in comparison to the effects of extreme Syx5 inhibition found in previous studies [1], [2]. Early endosome localization: Immunocytochemistry in GM2069 cells utilized anti-Syx5 (1∶50) and mouse anti-EEA1 was used as an early endosome marker (1∶100, BD Biosciences). Secondary antibodies, DAPI staining and image analysis were conducted as described above for Golgi distribution. GM2069 cells were treated with control siRNA (G–I) or Syx5 siRNA (J–L). Syx5 is shown in Green (G, J), EEA1 is shown in Red (H, K) and DAPI is shown in Blue. Merged images are also shown (I, L). The localization of early endosomes was not affected by Syx5 suppression (J–L). 1. Amessou M, Fradagrada A, Falguieres T, Lord JM, Smith DC, Roberts LM, Lamaze C, Johannes, L [file pone.0014303.s008.tif]
